# Aquaporin-4 reduces neuropathology in a mouse model of Alzheimer’s disease by remodeling peri-plaque astrocyte structure

**DOI:** 10.1186/s40478-019-0728-0

**Published:** 2019-05-08

**Authors:** Alex J. Smith, Tianjiao Duan, Alan S. Verkman

**Affiliations:** Departments of Medicine and Physiology, University of California San Francisco, HSE 1246, 513 Parnassus Ave, San Francisco, CA 94143 USA

**Keywords:** Astroglia, Water channel, Neurodegeneration, Glial barrier

## Abstract

Redistribution of the water channel aquaporin-4 (AQP4) away from astrocyte endfeet and into parenchymal processes is a striking histological feature in mouse models of Alzheimer’s disease (AD) and other neurological conditions with prominent astrogliosis. AQP4 redistribution has been proposed to impair bulk Aβ clearance in AD, resulting in increased amyloid deposition in the brain; however, this finding is controversial. Here, we provide evidence in support of a different and novel role of AQP4 in AD. We found that *Aqp4* deletion significantly increased amyloid deposition in cerebral cortex of 5xFAD mice, with an increase in the relative number of fibrillar vs. dense core plaques. AQP4 deficient 5xFAD mice also showed a significant reduction in the density of GFAP labeled peri-plaque astrocyte processes. Microglial plaque coverage was also significantly reduced, suggesting astrocyte involvement in organizing the peri-plaque glial response. The alterations in peri-plaque glial structure were accompanied by increased neuronal uptake of Aβ and an increase in the number of dystrophic neurites surrounding plaques. On the basis of these findings, we propose that redistribution of AQP4 into the parenchymal processes facilitates astrocyte structural plasticity and the formation of a reactive glial net around plaques that protects neurons from the deleterious effects of Aβ aggregates. AQP4 redistribution may thus facilitate plaque containment and reduce neuropathology in AD.

## Introduction

In healthy brain the water channel aquaporin-4 (AQP4) is heavily enriched at the endfeet of astrocytes in the form of supramolecular aggregates that appear as orthogonal arrays in freeze-fracture electron micrographs of the endfoot membrane [38, 48]. AQP4 is upregulated and redistributed in reactive astrocytes, and becomes prominently expressed in parenchymal astrocyte processes in rodent models of many neurological diseases including Alzheimer’s Disease (AD) [50, 56]. The significance of AQP4 redistribution in AD is uncertain. One explanation has been offered, a ‘glymphatic’ hypothesis proposing that mislocalization of AQP4 causes failure of a trans-endfoot convective fluid flow, which impairs clearance of toxic protein aggregates from the brain parenchyma into the peri-venular spaces during sleep [23, 54]. In support of this hypothesis, an increase in β-amyloid deposition has been observed in APP/PS1 mice that lack AQP4 [55], and human *AQP4* polymorphisms have been reported as a genetic risk factor for AD [7, 37]. However, the plausibility of the ‘glymphatic’ hypothesis has been questioned on theoretical grounds [2, 21, 24] and from the fact that clearance of β-amyloid aggregates occurs primarily via the peri-arterial and not the peri-venular spaces [1, 8]. Further, the relevance of the glymphatic hypothesis to human disease is unclear due to differences in AQP4 distribution between rodents and humans [16], and in independent studies we did not observe AQP4-facilitated parenchymal convection [44]. Therefore, alternative mechanisms may be needed to explain the deleterious effect of *Aqp4* deletion in experimental AD models.

Astrogliosis is a pathological hallmark of AD with complex effects on disease progression [4, 12, 49]. Reactive astrocytes phagocytose amyloid aggregates and dystrophic synapses [19, 34, 52], form a reactive glial net that surrounds and invades plaques [6], and are involved in the inflammatory response to amyloid deposition [53]. AQP4 facilitates astrocyte migration and glial scar formation in the response to traumatic brain injury [3, 39] and is involved in astrocyte cytokine release in response to neuroinflammation [30]. AQP4 function as an effector in the astrocyte response to brain injury suggests that its redistribution to peri-plaque astrocyte processes might be an important component of the astrocyte response in AD, rather than a pathological consequence of endfoot damage.

Here, we compared amyloid deposition and peri-plaque astrocyte structure in the 5xFAD mouse model of AD [35] bred with *Aqp4* knockout mice. We found increased amyloid deposition in *Aqp4* knockout 5xFAD mice compared with wild-type 5xFAD littermates, with marked alterations in plaque structure, peri-plaque astrocyte and microglial organization, and neuronal injury surrounding plaques. These results demonstrate that parenchymal AQP4 participates in peri-plaque astrocyte structural reorganization, and support a novel role for AQP4 in AD pathogenesis in which its redistribution facilitates plaque containment and reduces neuropathology.

## Materials and Methods

### Mice

5xFAD mice overexpressing human APP and PSEN1 with AD-associated mutations were obtained from the NIH mutant mouse research and resource center (MMRCC). These mice were bred with *Aqp4*^*−/−*^ mice on a C57Bl/6 background that were previously generated in our laboratory [32]. Offspring were genotyped for APP and PSEN1 (which co-segregated as expected) and for *Aqp4*. F1 offspring that were heterozygous at all 3 loci (5xFAD^+/−^; *Aqp4*^+/−^) were then interbred and F2 offspring were genotyped as before. F2 offspring that were heterozygous for APP and PSEN1 and either wild-type or homozygous negative for *Aqp4* (5xFAD^+/−^; *Aqp4*^+/+^ or 5xFAD^+/−^; *Aqp4*^−/−^) were maintained until 7–9 months of age and then sacrificed by transcardial perfusion with 4% formaldehyde and the brain was processed for paraffin embedding. All procedures were approved by the UCSF Institutional Animal Care and Use Committee. A total of 5 *Aqp4*^−/−^ 5xFAD mice and 8 wild-type littermates were used in this study.

### Human samples

Tissue was obtained from the UCSF neurodegenerative disease brain bank, 3 AD and 3 control samples were studied. AD samples (2 M, 1F) had both clinical and neuropathological diagnosis of AD without comorbidity, mean age at death was 84 years and the post-mortem interval before fixation was 5.6–8.2 h. Control samples (1 M, 2F) had no clinical neurological diagnosis, 1 sample had no post mortem neuropathological diagnosis, 1 had a pathological diagnosis of cerebrovascular disease, and 1 was diagnosed with Lewy Body Disease in the brainstem. Mean age at death was 86.7 years and the post mortem interval was between 7.8–9.8 h.

### Antibodies

The following primary antibodies were used in this study: rabbit polyclonal anti-AQP4 (Sigma, cat # SAB5200112), goat polyclonal anti-AQP4 (Santa Cruz, cat # sc9888), chicken polyclonal anti-GFAP for staining astrocyte processes (Millipore, cat # AB5541), mouse monoclonal 6E10 anti-Aβ (Eurogentec, cat # SIG-39320), rabbit polyclonal Iba-1 for staining microglia (Wako, cat # 019–19,741), rabbit polyclonal anti-NeuN for staining neurons (Millipore, cat # ABN78), and rabbit monoclonal anti-synaptophysin for identification of pre-synaptic dystrophies (Abcam, cat # ab52636). Alexa 488, 555 or 647 conjugated secondary antibodies (Invitrogen) were used for detection.

### Immunostaining

5-μm thick microtome sections were cut from paraffin-embedded samples and mounted on glass slides. Sections were allowed to dry for at least 48 h after sectioning, then deparaffinized with xylene and rehydrated in a dilution series of water in ethanol. Antigen recovery was performed by placing slides in boiling 10 mM citrate, 0.05% Tween-20, pH 6.0 and allowing them to cool for 30 min. Slides were then equilibrated in PBS and blocked in PBS containing 1% BSA and 0.1% Triton X-100 for 30 min. Slides were stained with primary antibodies in 1 μg/ml in blocking buffer for 1 h at room temperature, rinsed 3 times with PBS, then stained with secondary antibodies at 1 μg/ml in blocking buffer. Slides were then washed 5 times in PBS with a 5 min interval for each wash, then mounted under coverslips in ProLong Gold antifade. For thioflavin S staining, samples were incubated in a 1% solution of thioflavin S in PBS for 5 min following antibody staining. Samples were then washed 5 times in PBS with a 5 min incubation between each wash, prior to mounting.

### Microscopy and image analysis

Sections were imaged with a Nikon C1 confocal microscope using 4x NA0.15, 20x NA0.5 or 100x NA1.4 objectives. For 3-color imaging, samples were scanned sequentially with the 488 nm, 561 nm and 647 nm lasers to ensure minimal crosstalk between channels. Analysis of plaque size and number was done using FIJI software. 20x images of Aβ staining from brain cortex were intensity-thresholded and objects greater than 50 μm^2^ were counted and measured for size. Plaque morphological analysis was done by comparing the relative staining intensity of immunolabeled Aβ and thioflavin S. For measurement of the density of glial processes in and around plaques, 100x images of GFAP and Aβ labeled sections were used. Aβ images were blurred with a Gaussian filter of 2 μm radius and thresholded to define the plaque boundary. A peri-plaque region extending 5 μm around the plaque was then created using the ‘*make band’* function in FIJI. The average GFAP intensity in these 2 areas was then determined for each plaque and normalized to the average GFAP intensity in the surrounding field of view. The number of Iba1-positive cells was determined by intensity thresholding and automated counting; for measurement of plaque microglial coverage, the plaque perimeter and the length of microglial processes associated with the perimeter were measured manually. For measurement of neuronal Aβ uptake, the fraction of NeuN-positive cells containing 3 or more Aβ labeled puncta surrounding plaques was calculated. For measurement of AQP4 polarization, the background corrected mean fluorescence intensity of AQP4 labeling was determined within manually drawn ROIs in the perivascular or peri-plaque regions and divided by the mean intensity within non-plaque parenchymal areas.

### Super-resolution microscopy

dSTORM imaging of paraffin sections from 5xFAD mice, where astrocyte processes were labeled with anti-GFAP antibody and Alexa 647 labeled secondary antibody, was performed as described previously [43].

### Statistical analysis

Data was collated in Microsoft Excel. Statistical tests and graphing were performed with GraphPad Prism v. 5.01.

## Results

### Increased plaque size but reduced compact amyloid in 5xFAD mice lacking AQP4

**Fig. 1:**
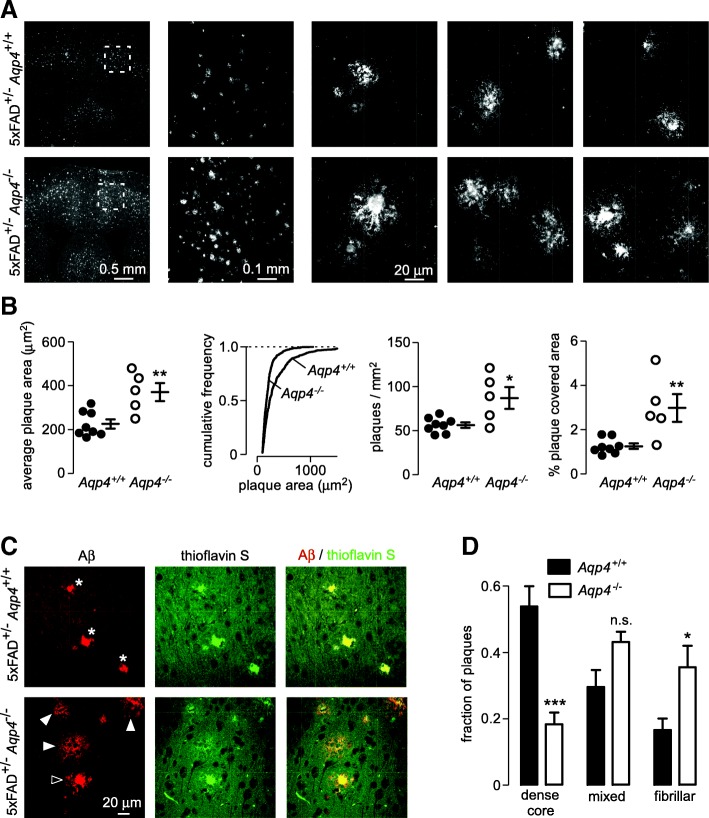
Increased amyloid accumulation and more fibrillar plaques in *Aqp4* deficient 5xFAD mice. **a** Distribution of immunolabeled Aβ plaques in the brain of *Aqp4* deficient and wild-type 5xFAD littermate at low and intermediate magnifications, and representative high magnification images of individual plaques. **b** Average plaque size in each mouse (left panel), cumulative plaque size distribution of all amyloid plaques in 5xFAD mice of each genotype, (center left panel), density of plaques in cortex (center right panel) and total amyloid load (right panel), as determined by intensity thresholding and automated object counting. * *p* < 0.05, ** *p* < 0.01 by unpaired t-test. Lines show mean and S.E.M. of each genotype, *n* = 8 *Aqp4*^+/+^ and 5 *Aqp4*^−/−^ mice. **c** Aβ immunolabeling and thioflavin S staining showing dense core (asterisk), fibrillar (solid arrow) or mixed (open arrow) plaques. **d** Fraction of plaques in each class. *** *p* < 0.001, *p < 0.05, n.s *p* > 0.05 by 2-way ANOVA with Bonferroni post-test

To investigate the effect of *Aqp4* deletion on amyloid deposition in the brain we generated 5xFAD mice that lacked *Aqp4* and aged them for 7–9 months before sacrifice. Staining of coronal sections with antibodies to Aβ revealed increased amyloid deposition in *Aqp4* deficient 5xFAD mice (Fig. 1a). To quantify amyloid deposition, intensity-based thresholding and automated object counting were used to compare the size and number of plaques in individual mice. Analysis of the average plaque size and frequency distribution of plaque sizes showed significantly greater plaque size in the absence of AQP4 (Fig. 1b left and center left panels). The number of plaques in cerebral cortex was significantly greater in the *Aqp4* deficient 5xFAD mice, which in combination with increased plaque size resulted in a more than 2-fold increase in total amyloid deposition (Fig. 1b center right and right panels).

Amyloid plaques are classically described as dense-cored, fibrillar or diffuse based on morphological criteria and affinity for dyes that bind β-pleated sheets such as thioflavin S [10, 14]. We found that plaques from 5xFAD mice could be classified as dense-cored, fibrillar or mixed (compact core with fibrillary ‘halo’) with compact plaque cores showing very bright thioflavinS labeling and fibrillary plaques showing weaker thioflavin S labeling. Classical diffuse plaques, which have no thioflavinS labeling and no associated neuritic damage, were not seen in the 5xFAD mice. We found significantly more fibrillar plaques and significantly fewer dense core plaques in the *Aqp4* knockout 5xFAD mice (Fig. 1d), indicating an altered amyloid accumulation pattern in the absence of AQP4.

### AQP4 is required for enrichment of astrocyte processes within and around plaques

**Fig. 2:**
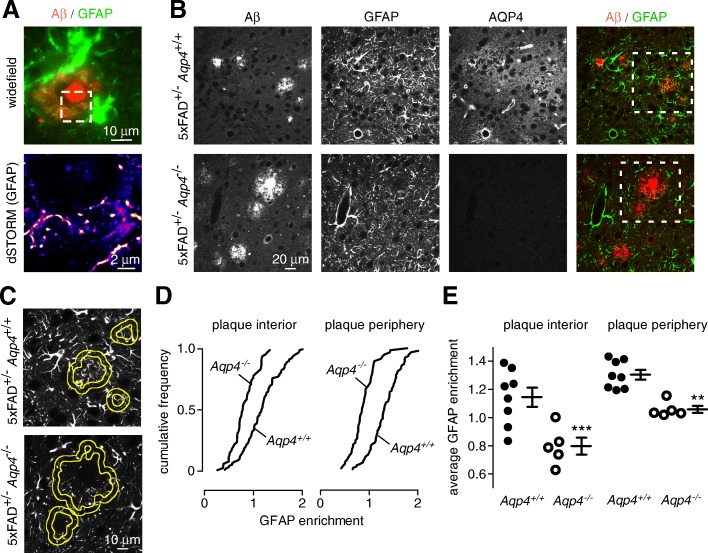
Reduced peri-plaque astrocyte coverage in AQP4 deficient 5xFAD mice. **a** Super-resolution imaging demonstrates a mesh of GFAP labeled processes surrounding the plaque core in a wild-type 5xFAD mouse. **b** Aβ, GFAP and AQP4 immunofluorescence showing astrocyte processes around and within plaques of *Aqp4*^+/+^ and *Aqp4*^−/−^ 5xFAD mice. The dotted square area denote the region shown in **c**. **c** Boundaries of segmented plaque interior and plaque periphery ROIs (yellow) superimposed on the corresponding GFAP image of astrocyte processes used to determine the extent to which astrocytes surround and infiltrate plaques. **d** GFAP enrichment, defined as average GFAP intensity within the indicated area divided by average GFAP intensity in the surrounding field of view, from within (‘plaque interior’) and around (‘plaque periphery’) plaques displayed as the cumulative frequency for all plaques from *Aqp4*^+/+^ and *Aqp4*^−/−^ 5xFAD mice. **e** Average GFAP enrichment within and around plaques for all measured plaques in each individual *Aqp4*^+/+^ or *Aqp4*^−/−^ 5xFAD mouse (*Aqp4*^+/+^ *n* = 8, *Aqp4*^−/−^ *n* = 5; ^**^ *p* < 0.01, *** *p* < 0.001 by unpaired t-test)

Astrocytes and microglia form a reactive glial net around plaques [6]. By super-resolution microscopy a fine web of GFAP-labeled processes was seen surrounding plaque cores in wild-type 5xFAD mice (Fig. 2a). As AQP4 facilitates astrocyte migration and glial scar formation at sites of brain injury [39], we investigated the effect of *Aqp4* deletion on the arrangement of astrocyte processes surrounding plaques.

Immunofluorescence in Fig. 2b shows that GFAP-positive processes surround and invade plaques in wild-type 5xFAD mice; however, GFAP labeled processes were less prominent inside and around plaques in *Aqp4* knockout 5xFAD mice. The extent of GFAP labeling inside and around plaques was quantified by intensity thresholding to identify plaque and peri-plaque regions of interest (Fig. 2c). GFAP labeling was increased in both the plaque interior and peri-plaque regions in wild-type 5xFAD mice when compared with the remaining, non-plaque-containing field of view. In the absence of AQP4, the GFAP labeling intensity in both the plaque interior and peri-plaque regions was significantly reduced compared to the non-plaque area (Fig. 2d, e). These results suggest the involvement of AQP4 in remodeling astrocyte processes around amyloid plaques.

### Reduced microglial recruitment to plaques in AQP4 deficient 5xFAD mice

**Fig. 3:**
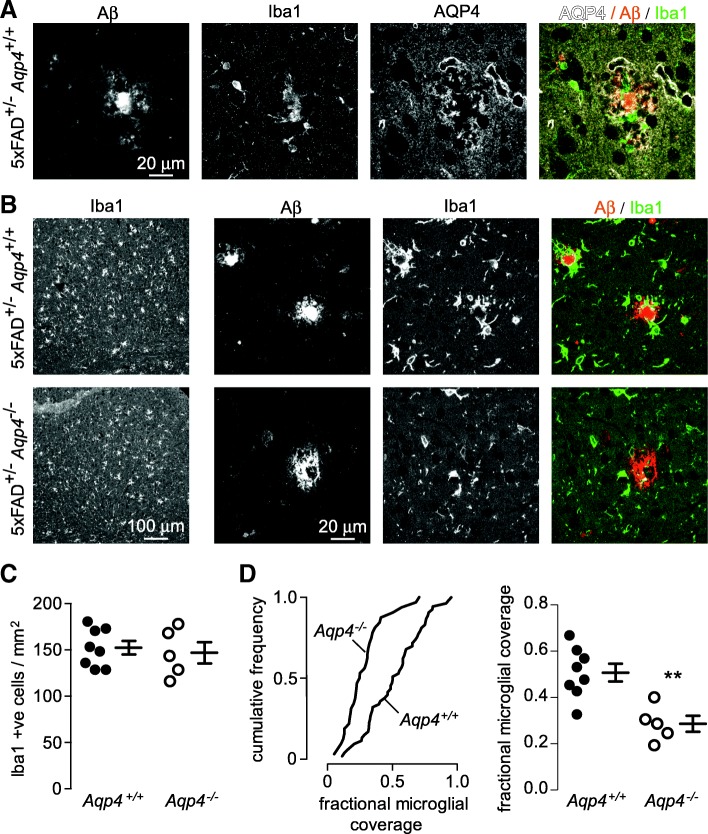
Reduced microglial plaque coverage in *Aqp4* deficient 5xFAD mice. **a** Aβ, Iba1 and AQP4 immunofluorescence showing close association peri-plaque AQP4-labeled astrocyte processes and Iba1-labeled microglia in wild-type 5xFAD mouse. **b** Low (left panels) and high (right panels) magnification images of Iba1-labeled microglia surrounding plaques in *Aqp4*^+/+^ and *Aqp4*^−/−^ 5xFAD mice. **c** Average number of Iba1-expressing cells in cortex of *Aqp4*^+/+^ and *Aqp4*^−/−^ 5xFAD mice as determined by thresholding and object counting (difference not significant). **d** Fractional coverage of plaque cores with Iba1-labeled microglial processes displayed as a frequency distribution for all plaques from *Aqp4*^+/+^ and *Aqp4*^−/−^ 5xFAD mice (left panel). Average extent of plaque containment by microglia as determined for at least 10 plaques in each individual mouse (right panel). (*Aqp4*^+/+^ *n* = 8, *Aqp4*^−/−^ *n* = 5, **p < 0.01 by unpaired t-test)

Motivated by the importance of microglia in degrading Aβ [28] and forming compact plaque cores [13, 57], we investigated the effect of *Aqp4* deletion on the microglial response in 5xFAD mice. Immunofluorescence with antibodies to Aβ, Iba1 and AQP4 from a wild-type, 5xFAD mouse showed AQP4-enriched astrocyte processes surrounding both plaque and plaque-associated microglia (Fig. 3a). Iba1 staining in wild-type and *Aqp4* deficient 5xFAD mice was compared to determine if *Aqp4* deletion disrupted peri-plaque microglial organization. Low magnification images (Fig. 3b left; Fig. 3c) show similar numbers of Iba1-positive cells in wild-type and *Aqp4* deficient 5xFAD mice. At higher magnification, microglia in wild-type mice were seen to surround and contain plaques either completely or partially; however, microglia did not form an effective barrier around plaques in *Aqp4* deficient 5xFAD mice (Fig. 3b). The fraction of the plaque perimeter bounded by microglia was significantly reduced in *Aqp4* deficient 5xFAD mice (Fig. 3d).

### Increased intraneuronal Aβ in AQP4 deficient 5xFAD mice

**Fig. 4:**
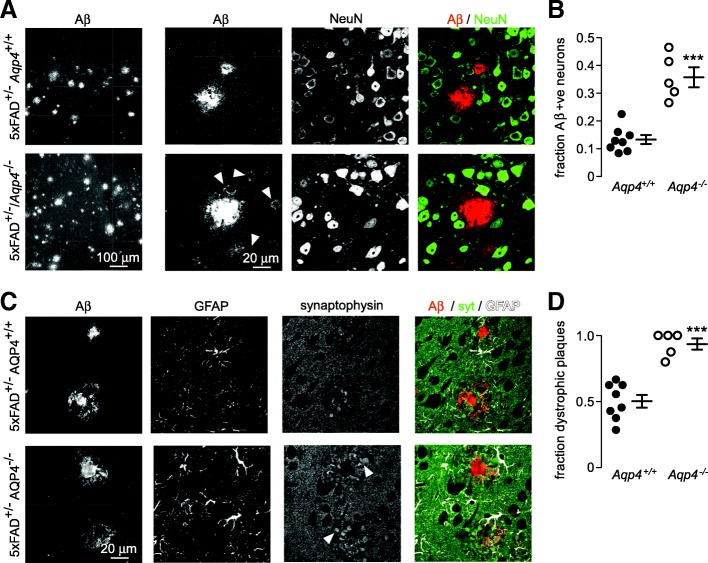
Increased neuronal Aβ uptake and more peri-plaque dystrophic neurites in *Aqp4* deficient 5xFAD mice. **a** Intermediate (left panels) and high (right panels) magnification images showing Aβ uptake in NeuN-labeled neurons surrounding plaques (arrowheads). **b** The fraction of Aβ-positive neurons surrounding plaques as measured in at least 10 separate plaques from each mouse of each genotype (*Aqp4*^*+/+*^ *n* = 8, *Aqp4*^*−/−*^ *n* = 5, *** *p* < 0.001 by unpaired t-test). **c** Synaptophysin labeled presynaptic dystrophies (arrowheads) surrounding plaques showing increased peri-plaque dystrophies in *Aqp4* deficient 5xFAD mice. **d** The fraction of dystrophic plaques (4 or more large presynaptic dystrophies) determined in 8–10 plaques from each mouse (*Aqp4*^*+/+*^ *n* = 8, *Aqp4*^*−/−*^ *n* = 5, *** *p* < 0.001 by unpaired t-test)

Accelerated cognitive decline has been reported in AQP4 deficient APP/PS1 mice [55] and intraneuronal Aβ deposits are associated with neuron loss and cognitive deficit [11, 51]. Extensive uptake of Aβ by cells in the vicinity of plaques was visible in *Aqp4* deficient 5xFAD mice (Fig. 4a left panels, arrowheads), which by co-staining with the neuronal marker NeuN identified these cells as neurons (Fig. 4a, right). The fraction of neurons around plaques with punctate Aβ staining was markedly increased in *Aqp4* deficient 5xFAD mice (Fig. 4b). The formation of dystrophic neurites around plaques is another feature of Aβ mediated neurotoxicity [18]. The density of presynaptic dystrophies surrounding plaques was visualized using the synaptic vesicle marker synaptophysin [25, 40] (Fig. 4c, arrowheads). The fraction of neuritic plaques containing four or more large presynaptic dystrophies was much greater in the absence of AQP4 (Fig. 4d), further supporting the conclusion that disorganization of the peri-plaque glial net results in increased toxicity to surrounding neurons.

### AQP4 distribution in human AD

**Fig. 5:**
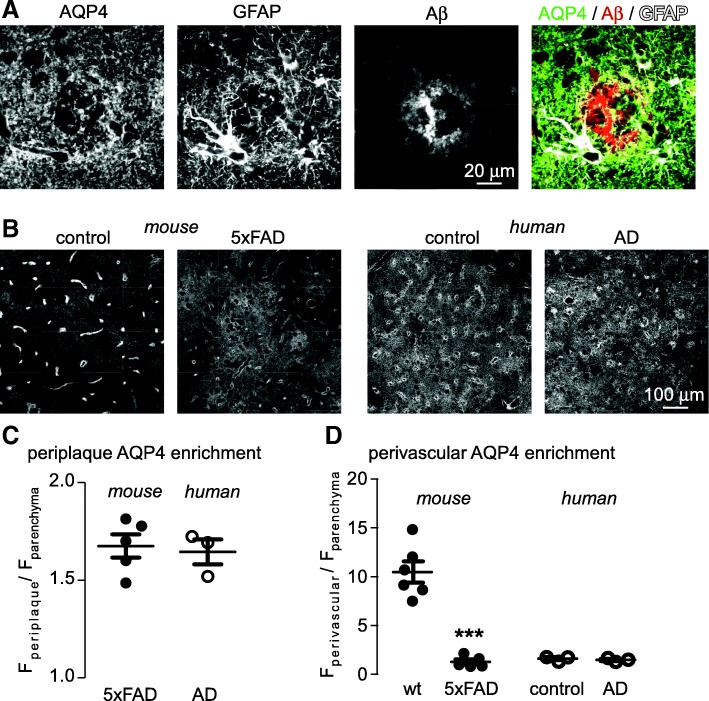
AQP4 is increased in peri-plaque astrocyte processes in post-mortem samples from human AD patients. **a** AQP4, GFAP and Aβ immunofluorescence in inferior frontal gyrus of a human AD patient showing AQP4 enrichment in peri-plaque astrocyte processes. **b** Lower magnification images showing extensive redistribution of AQP4 away from endfeet in 5xFAD mice compared to age matched wild-type mice, and corresponding images from aged control and AD human patient samples, demonstrating that AQP4 is mostly parenchymal in both cases. **c** Average enrichment of AQP4 in peri-plaque astrocyte processes, as compared to non-plaque parenchymal areas, in mouse and human AD. **d** Average enrichment of AQP4 in perivascular regions, compared to the parenchyma, in mouse and human control and AD. Samples from 6 wild-type and 5 5xFAD mice were compared along with 3 control samples and 3 AD human samples. (*** *p* < 0.001 by unpaired t-test)

Redistribution of AQP4 away from endfeet has been reported in rodent models of AD [50, 56]; however, in human brain the basal polarization of AQP4 to endfeet is far less dramatic and only small disease-associated changes in endfoot AQP4 have been suggested [16, 58]. We investigated AQP4 distribution in post-mortem samples of the inferior frontal gyrus from patients with diagnosed AD and age-matched controls without AD. In the AD brain, AQP4 was enriched in the peri-plaque processes of reactive astrocytes (Fig. 5a, c), as was observed in mice (Fig. 2a). In rodent AD, AQP4 was clearly redistributed away from endfeet (Fig. 5b, left, Fig. 5d); however, this was not observed in human brain, where AQP4 was not as heavily polarized in control samples (Fig. 5b right). These results demonstrate that the enrichment of AQP4 in peri-plaque astrocyte processes as described herein is relevant to human AD; however, the dramatic redistribution of AQP4 away from endfeet observed in mouse AD models is not observed in human, due to lower basal AQP4 levels in endfeet.

## Discussion

**Fig. 6:**
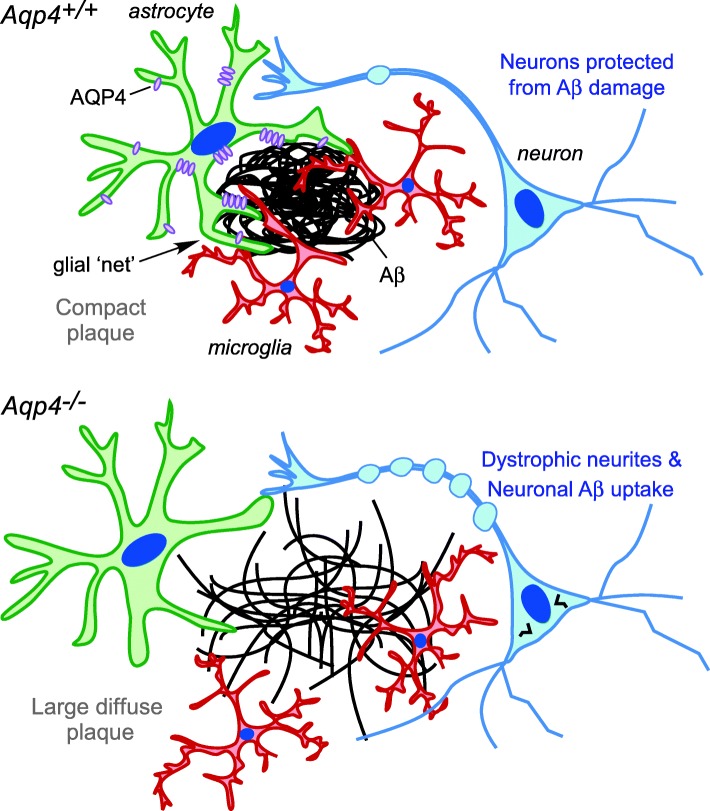
Proposed role of AQP4 in containment of amyloid plaques and recruitment of microglia. When AQP4 is present, its redistribution to peri-plaque astrocyte processes is associated with formation of a glial net surrounding the plaque, recruitment of microglia, and partial protection of nearby neurons from the deleterious effects of Aβ aggregates. In AQP4 deficiency, astrocytes fail to contain plaques or recruit microglia, resulting in large fibrillar plaques that cause greater damage to nearby neurons

Our data here that *Aqp4* deletion increases amyloid deposition in a mouse model of AD is in agreement with prior reports [55], but challenge the prevailing notion that the increased amyloid is a consequence of reduced Aβ clearance due to loss of perivascular AQP4 [22]. Instead, we found that *Aqp4* deletion markedly impairs peri-plaque astrocyte structural organization and the recruitment of microglia to plaques. This is associated with an increase in the number of fibrillar plaques and consequent greater damage to neurons surrounding plaques, which can account for the reported worsening of cognitive signs in *Aqp4* deficient AD model mice [55]. On the basis of these results we propose a novel mechanistic model to account for the role of AQP4 in amyloid accumulation (Fig. 6). According to this model, increased expression of AQP4 in glial processes occurs in response to the initial formation of insoluble amyloid aggregates, which facilitates structural rearrangements in astrocytes and the recruitment of microglia to plaques. The resulting physical barrier helps to segregate plaques from the surrounding tissue.

An increase in amyloid plaque accumulation in AD can potentially be attributed to impaired Aβ clearance, increased Aβ production, or a greater propensity for soluble Aβ to incorporate in plaques. Clearance of Aβ monomers may occur by degradation (25–50%), transport across the blood brain barrier (25–40%), or bulk clearance into the CSF (~ 10%) [9, 20, 45]. Iliff et al. [23] reported that clearance of injected Aβ from the brain was sensitive to *Aqp4* deletion, and proposed a role for AQP4 in bulk clearance. However, the time course of clearance was more rapid than that of other markers such as inulin that are cleared by a bulk clearance mechanism [20]. We did not find significantly altered distribution of injected Aβ in *Aqp4* deficient mice [44], which suggests that increased amyloid accumulation in *Aqp4* deficient mice is unlikely to be explained by impaired clearance. Since presynaptic dystrophies are major sites of Aβ synthesis [40], the increased number of dystrophic neurites in *Aqp4* deficient AD mice may increase amyloid accumulation and neuronal Aβ uptake. Additionally, incorporation of soluble Aβ into plaques is increased in regions that are not surrounded by glia [13]. Finally, plaque morphological alterations are also potentially a consequence of failure of peri-plaque glia to condense amyloid [57], although other explanations are possible.

We find that the density of astrocyte processes surrounding and within amyloid plaques is reduced in *Aqp4* deficient AD mice. The presence of astrocyte processes in amyloid plaques from AD patients has been long recognized [15] and the barrier formed by astrocytes and microglia around plaques was recently described as a reactive glial net [6]. Deletion of astrocyte intermediate filament proteins that are involved in structural reorientation increases amyloid accumulation in mouse models of AD [26], suggesting a role for astrocyte structural reorganization in limiting amyloid accumulation. In addition to GFAP and AQP4, peri-plaque astrocyte processes are enriched in connexins [33], suggesting structural similarities to other glial barriers such as the glia limitans and glial scars formed following trauma. The observation here that AQP4 participates in reorientation of astrocytic processes toward plaques is consistent with previous observations that AQP4 facilitates extension of astrocyte processes during their migration [42] and glial scar formation in response to trauma [39]. Mechanistically, this is thought to occur via coupling of solute and water uptake, which osmotically inflates the leading edge of extending processes and facilitates actin polymerization-driven extension [36].

An additional finding was impaired recruitment of microglia to plaques in *Aqp4* deficient AD mice. Microglia phagocytose and degrade Aβ [28] and deletion of microglial chemokine receptors prevents microglial recruitment to plaques and increases amyloid deposition [17], similar to the changes observed here in *Aqp4* deficient AD mice. Astrocytes are an important source of inflammatory cytokines in AD [29] and AQP4 deletion impairs release of proinflammatory cytokines by astrocytes [30, 31], suggesting that the failure of microglial accumulation around plaques in *Aqp4* deficient AD mice might be a consequence of impaired astrocyte cytokine release. Although the mechanism of AQP4-dependent cytokine release is not known, *Aqp4* deletion impairs astrocyte Ca^2+^ signaling [5, 47], which is involved in cytokine release [41]. Astroglial ApoE and liver X receptor are required for microglial Aβ phagocytosis [46], providing further evidence that interaction between astrocytes and microglia is required for efficient Aβ degradation. Also, perhaps altered plaque structure and/or failure to remodel the extracellular space in *Aqp4* deficient mice could lead to failure of microglia to interact with plaques.

*Aqp4* deletion was associated with an increase in the number of plaques surrounded by dystrophic neurites and an increase in the number of neurons containing Aβ aggregates. Although the cause of this is unclear, it may be related to impairment in plaque containment or to the greater amount of aggregated amyloid present in the mice lacking *Aqp4*. Neuritic damage associated with fibrillary plaques may be due to direct penetration of the neuronal membrane by extending amyloid fibrils in the absence of glial containment [13, 57]. Reactive astrocytes also play an important role in phagocytosis and degradation of dystrophic neurites. Since AQP4 is heavily enriched in the limiting membrane of astrocyte phagosomes [19], *Aqp4* deletion may also impair clearance of damaged neurites from the peri-plaque area.

The marked enrichment of AQP4 in astrocyte endfeet and its redistribution to the parenchyma in mouse models of AD have led to speculation that loss of endfoot AQP4 may be an important cause of amyloid accumulation in the ageing brain due to impairment of the ‘glymphatic’ system [27]. However, the extent of AQP4 enrichment in endfeet in human brain is much less than in rodents [16] and the extent of AQP4 redistribution in human AD is very limited [58], calling into question the relevance of findings from mouse models to human AD. In agreement with these findings we were unable to find any indication of AQP4 redistribution from endfeet to the non-plaque parenchyma in a limited number of human AD samples. We did, however, find that AQP4 was enriched within the peri-plaque glial net surrounding plaques from human AD patients to the same extent as in the mouse model, suggesting that AQP4 mediated remodeling of peri-plaque astrocyte processes is relevant to the human disease.

## Conclusions

In summary, our results support a novel role for AQP4 in the pathology of AD and highlight the importance of the peri-plaque glial environment as a determinant of amyloid neurotoxicity. Further understanding of the role of astrocyte water transport in formation of glial barriers is therefore expected to identify novel therapeutic approaches that can enhance endogenous protective mechanisms that limit amyloid neuropathology in AD.

## Abbreviations

5xFAD: Mouse containing transgenes with 5 familial Alzheimer’s disease mutations
AD: Alzheimer’s disease
APP: Amyloid precursor protein
*AQP4*: Aquaporin-4 (human gene)
*Aqp4*: Aquaporin-4 (mouse gene)
AQP4: Aquaporin-4 (protein)
Aβ: β amyloid
BSA: Bovine serum albumin
dSTORM: direct stochastic optical reconstruction microscopy
GFAP: Glial fibrillary acidic protein
MMRC: Mutant mouse resource center
PBS: Phosphate buffered saline
PSEN1: Presenilin 1
ROI: Region of interest
